# Correspondence

**Published:** 1875-11

**Authors:** 


### Correspondence.

QUINCY, ILL., July 19th, 1875.

EDITOR CHICAGO MEDICAL JOURNAL AND EXAMINER—

DEAR SIR:—In the *American Journal of the Medical Sciences* for this month, in an article on "Ununited Fractures," by Dr. John H. Packard, of Philadelphia, reference is made to a case reported by Mr. Annandale in the *British Medical Journal* for January 9th, 1875, and subsequently reported in the *Monthly Abstract of Medical Science*, March, 1875, page 127.

A case of Colles' Fracture, resulting in great deformity of the forearm, occurred in this city eight years ago; the operation of resection subsequently performed for its relief by Dr. William A. Byrd, very closely resembling that which Dr. Packard describes in the first of his two cases. Dr. Byrd's operation was performed November 1st, 1873, and an account of it was published, with plates, in the *Richmond and Louisville Medical Journal*, for October, 1874.

The *Monthly Abstract* for March, 1875, contains Mr. Annandale's report, but not until the April number of that publication, and then only when the attention of the editor had been called to the matter, was Dr. Byrd's report noticed. Now comes Dr. Packard, who refers again to Annandale, but seems to have no knowledge whatever of the operation performed and reported by an American surgeon several months before. What is the reason for all this? Do our brethren in the East imagine that in the East only can wisdom be found? Is it not possible at least, that had the case reported by Dr. Byrd occurred in one of the Atlantic States (or say in the city of Philadelphia), Dr. Packard would have remembered something about it, and Dr. Hays would not have omitted and utterly ignored it, in making up his *Abstract*, until in some sort compelled to give it the notice it deserved?

As a friend of Dr. Byrd, and having been present at the operation, which was certainly one that had no precedent in medical literature, I send you these statements and queries which you are at liberty to use as you may see fit.

Philadelphia is, of course, entitled to her station as the old centre of medical science in this country ; but it behooves the priests around her altars, if they would uphold the sacred honor of their temple, to accord a portion of that honor, and to give credit to those entitled thereto, and not to cross the ocean in search of prophets, or to look for information that they have previously received from their own countrymen, even when it does come from that West over which they affect so great a superiority.

I am very truly yours,

L. H. COHEN, M.D.

---

New York, 43 W. 32nd St., Sept. 22, 1875.

My Dear Doctor—

In the last number of your Journal and Examiner, Dr. Leonard, of Detroit, writes upon the use of warm and hot water in surgery; "not so much," as he says, "to call attention to the efficacy of this plan of treatment, as to set aright some of the current opinions as to its originator." In the next paragraph, alluding to one of my own contributions upon the subject, he says : "Therein it seems to be fathered upon the French Surgeon, Amussat."

If Dr. Leonard will read the paper alluded to again, he will see that I have stated exactly the contrary ; namely, that Amussat had reached the conclusion that cool water was in general to be preferred.

The error was, of course, unintentional, but I have thought it of sufficient importance to require correction.

Very truly yours,

FRANK H. HAMILTON.

Wm. H. Byford, M.D.

